# Maternal *RFC1* Gene Polymorphisms and Neural Tube Defects: A Case–Control Study in Ethiopia

**DOI:** 10.3390/genes17040478

**Published:** 2026-04-17

**Authors:** Hasset Tamirat Molla, Dawd Gashu, Barbara Stoecker, Winyoo Chowanadisai

**Affiliations:** 1 Center for Food Science and Nutrition, Addis Ababa University, P.O. Box 1176, Addis Ababa 1000, Ethiopia; dawd.gashu@aau.edu.et; 2 Department of Nutritional Science, Oklahoma State University, Stillwater, OK 74078, USA; barbara.stoecker@okstate.edu (B.S.); winyoo.chowanadisai@okstate.edu (W.C.)

**Keywords:** single nucleotide polymorphism (SNP), neural tube defects (NTD), *RFC1* gene

## Abstract

Background: Etiologies of neural tube defects (NTDs) are multifactorial. Genetic, epigenetic and environmental factors may contribute to their reported variation in prevalence across the globe. Ethiopia has among the highest reported NTD prevalence globally, making investigation of genetic determinants in this high-risk population particularly important for advancing the understanding of NTD etiology. Genes involved in folate metabolism, such as the reduced folate carrier 1 (*RFC1*), have been investigated for the potential associations with NTDs, but findings throughout the literature remain inconsistent and inconclusive. Objective: The aim of this study was to determine an association of RFC-1 polymorphism at rs1131596 and rs1051266 loci (functional variants previously implicated in folate transport efficiency and NTD susceptibility) among mothers with the occurrence of NTDs in their offspring in Ethiopia. Methods: A case–control study involving 250 mothers (187 controls and 63 cases) of children with or without NTDs was conducted in Addis Ababa, Ethiopia from April 2022, to September 2024. A total of 250 maternal whole blood samples were systematically collected and subjected to genetic analysis at loci rs1131596 and rs1051266 by polymerase chain reaction (PCR) and Sanger sequencing. Results: Detection of heterozygous (TC) and homozygous (CC) genotypes for SNP rs1131596 (−43T>C) in the *RFC1* gene was 27.2%, with heterozygous (TC) comprising 10.4% and homozygous (CC) 16.8%. In contrast, for the rs1051266 (80A>G), the prevalence of the AG polymorphism was 28% while the GG polymorphism was 16.4%, resulting in a cumulative prevalence of 44.4%. The presence of maternal RFC-1 polymorphism at these two locations were not associated with significantly (*p* = 0.601 & *p* = 0.225 respectively) higher odds for NTD births. Conclusions: This study did not reveal significant association between maternal *RFC1* gene polymorphisms and NTD-affected births. Comprehensive whole-genome sequencing of affected off-spring is essential to identify specific mutations or polymorphisms that may individually or collaboratively affect the risk of NTDs in the Ethiopian context.

## 1. Introduction

Neural tube defects (NTDs) are structural defects of the central nervous system that affect the brain and spinal column during the first month after conception. NTDs occur due to the partial or complete failure of neural tube closure [1,2]. Ranging from mild to severe symptoms, the three main defects (anencephaly, encephalocele, and spina bifida) pose significant challenges. While anencephaly is typically associated with early mortality, individuals with spina bifida can survive with appropriate medical care, even though they often face substantial disability and limited life expectancy [3]. NTDs rank as the second most prevalent birth defect following congenital heart defects worldwide [4].

The global prevalence of neural tube defects is estimated at 18.6/10,000 (uncertainty interval: 15.3–23.0) live births, where approximately 75% of cases result in under-five mortality. An estimate of two cases per 1000 births would annually approximate 214,000–322,000 affected pregnancies worldwide [5].

Most of the mortality burden of NTDs is in low- and middle-income countries like Ethiopia. NTDs occur at a markedly higher rate in North Ethiopia compared to the global norm, with an estimated 131 affected infants per 10,000 births [6], in contrast to the worldwide average of 18.6 per 10,000 [5]. A recent study underscores the severity of the issue in Ethiopia, revealing the country’s high adjusted mortality fraction (7.5%) and adjusted mortality rate (104.0 per 10,000 births) attributed to NTDs if compared to South-East Asia (13.1 per 10,000 births) and other Sub-Saharan countries (14.2 per 10,000 births) [7]. Despite these alarming statistics, there is a significant gap in our understanding of the potential genetic risk factors associated with the occurrence of births affected with NTDs.

The etiology of NTDs is multifactorial, involving an interplay of genetic, epigenetic, and environmental determinants. Genetic contributions are estimated to account for up to 70% of the observed variance in NTD prevalence, highlighting their predominant role in disease susceptibility [8]. Among the genetic factors, genes involved in folate metabolism namely Reduced Folate Carrier 1 (*RFC1*) and Methyl Tetrahydro Folate Reductase (MTHFR) have been primarily indicated to have associations with births affected with NTDs with varying degrees of supportive evidence [9,10,11].

Case-control studies have found no significant association between the common *RFC1* A80G (rs1051266) polymorphism and NTD risk, suggesting that *RFC1* may not be a major determinant in folate-related NTD etiology across different populations [12,13]. In contrast, other investigations, particularly in populations with suboptimal folate status, have indicated a potential modest risk elevation linked to the G allele, possibly due to altered folate transport efficiency that impairs cellular folate availability during early embryogenesis [13,14].

The *RFC1* gene encodes a transmembrane protein crucial for transporting folate, which is essential for intracellular folate levels and normal cellular metabolism. Folate is a key precursor in purine and pyrimidine synthesis, necessary for DNA replication, repair, and methylation [14,15]. Variants like the A80G polymorphism in *RFC1* gene can reduce folate transport efficiency, leading to impaired nucleotide synthesis, disrupted methylation, and genomic instability. These effects are particularly critical during embryogenesis, increasing the risk of NTDs and other developmental abnormalities. Elevated homocysteine levels due to impaired folate metabolism may further contribute to oxidative stress and complications [13].

The importance of *RFC1* extends beyond metabolic pathways. The absorption of folate and its cellular uptake, facilitated by the *RFC1* gene encodes a cell surface transmembrane protein that facilitates the bidirectional movement of folate across cell membranes [12,16].

*RFC1* gene polymorphisms have been associated with several disease conditions including congenital anomalies like NTDs, ventricular septal defects (VSD), autistic spectrum disorders (ASD) [13], congenital heart disease (CHD) and orofacial defects [14]. The rs1131596 and rs1051266 SNPs within the *RFC1* gene have received attention due to their potential influence on *RFC1* function and one-carbon metabolism [12,13]. However, a conclusive understanding of the impact of these particular polymorphisms on NTD susceptibility remains elusive. In this study, we aimed to assess the contribution of *RFC1* polymorphisms at rs1131596 and rs1051266 in relation to the occurrence of NTDs within an Ethiopian population.

## 2. Materials and Methods

### 2.1. Study Setting

A hospital-based case–control study was conducted in two purposely selected hospitals (Zewditu Memorial Hospital and St. Peter’s Specialized Hospital) in Addis Ababa, Ethiopia. These two hospitals are among the leading referral hospitals in Addis Ababa with specialized pediatric neurosurgery departments. Infants and children affected by neural tube defects are referred to these centers from Addis Ababa and surrounding regions across Ethiopia. The study period was from April 2022 to September 2024.

Cases were defined as mothers of offspring affected by neural tube defects (NTDs), including major phenotypes such as spina bifida, anencephaly, and encephalocele, in accordance with standard classifications by the International Classification of Diseases (ICD-10). Hydrocephalus was not considered a primary NTD phenotype; however, cases of hydrocephalus secondary to NTDs (e.g., spina bifida with hydrocephalus) were included under the corresponding primary NTD diagnosis. Cases with ambiguous diagnoses, isolated hydrocephalus, hydrocephalus of non-NTD etiology, or multiple/non-NTD congenital anomalies were excluded.

### 2.2. Data Collection

Data collection for this study was done by trained General Practitioners (GPs) and nurses using a pretested questionnaire for comprehensive information gathering. These healthcare professionals interviewed mothers designated as cases and reviewed their medical records to confirm the diagnosis of NTDs. The absence of NTD anomalies was also explored in the control mothers’, i.e., mothers having normal children and visiting the hospitals for immunization or circumcision.

In parallel, whole blood samples from the mothers were collected by senior laboratory technicians following specialized training designed to ensure the consistency of sample collection. The data collectors utilized the Open Data Kit (ODKv.2023.1; (University of Washington, Seattle, WA, USA), which was installed on encrypted tablets for conducting interviews and recording responses. This approach was implemented to prioritize data integrity, privacy and security.

Participants were recruited using a consecutive sampling approach during the defined data collection period. All eligible mothers who attended the selected hospitals and met the inclusion criteria were invited to participate in the study until the end of the recruitment period. This approach allowed the inclusion of all available cases of mothers with NTD affected children presenting to the study sites, along with controls recruited from the same hospitals.

### 2.3. Ethical Approval and Consent for Research Participation

Ethical clearance was obtained from the National Research Ethics Review Board under the Ministry of Education, Ethiopia (IRB 17/246/818/22, 27 October 2022), from Addis Ababa University (IRB/02/14/2021, 28 September 2021) and from Oklahoma State University (IRB-24-234, 5 August 2024).

Participants were provided with an electronic information sheet and informed consent form using the ODK tool. Only individuals who willingly agreed to participate and formally signed the consent form were included in the research. The study was conducted in accordance with the ethical principles outlined in the Declaration of Helsinki.

### 2.4. Blood Collection for Genetic Analysis

Whole blood samples (3 mL) were systematically collected from mothers having infants and children affected with NTDs and from a control group of mothers with unaffected infants and children and stored in EDTA tubes. These blood samples were stored under −80 degrees in an EPHI laboratory until shipped to Oklahoma State University in the United States for genetic analysis. A total of 250 maternal samples were analyzed by PCR and DNA sequencing was done for *RFC1* single nucleotide polymorphisms (SNPs) rs1131596 and rs1051266.

### 2.5. DNA Extraction and Genotyping

Genomic DNA was extracted from 50 µL of whole blood using the HighPrepTM Blood & Tissue DNA Kit (MagBio Genomics, Gaithersburg, MD, USA) according to manufacturer’s instructions. Genomic DNA was stored at −20 °C until used for PCR. Genomic DNA was amplified by PCR using primers (5′-gtgaagttcttgtcgggccccaggaggtag-3′ and 5′-gcccagcagtgccatgagtctagtg-3′) designed by Primer3 software (version 4.1.0; Whitehead Institute for Biomedical Research, Cambridge, MA, USA) [17] and GoTaq Green polymerase (Promega, Madison, WI, USA). PCR conditions were as follows: an initial denaturation at 95 degrees for 2 min, then 35 cycles at 95 degrees for 30 s and annealing and extension at 68 degrees for 1 min (step 3), and a final extension at 72 degrees for 5 min. Samples were sequenced by Eurofins Genomics (Louisville, KY, USA) and chromatograms were viewed individually for *RFC1* SNPs rs1131596 and rs1051266, using methods similar to those published by our laboratory previously [18].

The selected SNPs (rs1051266 and rs1131596) in the RFC1 gene were chosen based on their reported functional relevance and prior evidence of association with folate metabolism and neural tube defects. In particular, the rs1051266 (80A>G) is a well-characterized functional polymorphism that may influence folate transport efficiency, while rs1131596 (−43T>C) has been investigated in previous association studies and may have regulatory significance [12,13,14].2.6. Statistical Analysis

Genotype and allele frequencies were calculated for both SNPs (rs1131596 and rs1051266), and HWE was assessed to evaluate genotype distribution within the study population. Minor allele frequency (MAF) was computed and compared with African population MAF data reported in the ALFA Allele Frequency Aggregator from the NCBI database [19,20]. Genotype distributions for each SNP were assessed for conformity with the Hardy–Weinberg equilibrium (HWE) in the control group using the chi-square test.

Associations between maternal genotypes and the risk of NTD-affected births were assessed using logistic regression. Regressions were applied under an additive genetic model, treating the number of minor alleles as a continuous predictor, and a recessive genetic model, comparing individuals homozygous for the minor allele with all others. Logistic regression analyses were conducted without adjustment for covariates to assess the direct relationship between genotype and outcome. In addition, a sensitivity analysis was performed to confirm that baseline characteristics did not act as confounding factors. All statistical analyses were conducted using SPSS version 24.0 (IBM Corp., Armonk, NY, USA), with statistical significance set at *p*-value less than 0.05.

## 3. Results

### 3.1. Participants’ Characteristics

The study enrolled 250 mothers with a case-to-control ratio of one-to-three. All cases were mothers of children diagnosed with spina bifida, a major phenotype of neural tube defects. Among these, 55.6% had spina bifida without hydrocephalus, while 44.4% had spina bifida with associated hydrocephalus (spina bifida with hydrocephalus). No cases of isolated (non-NTD-associated) hydrocephalus were included in the analysis. Among all participants 82.4% were in the age group of 20–34 years whereas 16% were beyond 35 years of age. The proportion of the mothers at the age of 35 and above constituted 17.5% of cases and 15.5% of controls (Table 1).

Among the study participants, 91.6% of the mothers were living in urban areas and 8.4% were from rural areas. Most of the participants were literate; 54.8% had completed high school and higher education and only 8% had no formal education. Nearly two thirds (62%) of the women were housewives.

### 3.2. Hardy–Weinberg Equilibrium (HWE) Analysis of RFC1 Polymorphisms

The genotype distributions of the two *RFC1* polymorphisms were assessed for compliance with Hardy–Weinberg equilibrium (HWE) in both case and control groups (Table 2). For rs1131596 (−43T>C), significant deviation from HWE was observed in both cases (χ^2^ = 25.39, *p* < 0.001) and controls (χ^2^ = 61.82, *p* < 0.001). Similarly, for rs1051266 (80A>G), the control group showed significant deviation from HWE (χ^2^ = 18.74, *p* < 0.001), whereas the case group did not deviate significantly (χ^2^ = 1.64, *p* = 0.127).

Overall, the observed genotype frequencies differed from those expected under HWE, particularly in the control group for both polymorphisms. These findings suggest potential influences such as population substructure or sampling variation, which should be considered when interpreting the genetic association results.

### 3.3. Comparison of RFC1 Allele Frequencies in Ethiopian and African Reference Populations

The allelic distribution of the selected *RFC1* polymorphisms among the study participants and their comparison with the broader African population averages are summarized in Table 3. For the rs1131596 (−43T>C) variant, the frequency of the reference (G) allele was notably higher in the Ethiopian cohort compared to the ALFA African average (0.719). Specifically, women with prior NTD births (cases) exhibited a mean allele frequency (MAF) of 0.818, while control women showed a MAF of 0.767.

Conversely, the alternate (A) allele frequency in the Ethiopian cases (0.182) and controls (0.232) was lower than the reported 1000 Genomes/ALFA African frequency of 0.280. Similarly, for the rs1051266 (80A>G) variant, the alternate (C) allele frequency in Ethiopian cases (0.238) was lower than both the Ethiopian controls (0.315) and the African reference population (0.327) [19,20].

### 3.4. RFC1 Gene Polymorphism (rs1131596 & rs1051266) Using the Additive Genetic Model

As shown in Table 4, under an additive genetic model, maternal *RFC1* polymorphisms were not significantly associated with NTD-affected births. For rs1131596, neither the heterozygous (TC) nor homozygous variant (CC) genotype showed a significant association compared to the TT reference genotype (TC: OR = 1.029, *p* = 0.953; CC: OR = 0.657, *p* = 0.325).

Similarly, for rs1051266, no statistically significant association was observed for either the AG genotype (OR = 1.144, *p* = 0.681) or the GG genotype (OR = 0.589, *p* = 0.248) compared to the AA reference group. Overall, no significant differences were observed in genotype distribution between mothers with and without a prior NTD-affected birth.

### 3.5. RFC1 Gene Polymorphism (rs1131596 & rs1051266) Using the Recessive Genetic Model

We investigated the role of rs1131596 & rs1051266 in *RFC1* gene further using a recessive genetic model, where mothers of homozygous carriers of the variant allele were compared with other genotypes combined (heterozygous and homozygous wild-type) to assess the potential association with the occurrence of NTD affected births.

For rs1131596 (−43T>C), the proportion of mothers with the homozygous variant genotype (CC) was lower among cases (12.7%) compared to controls (18.2%). Under the recessive model (CC versus TT + TC), logistic regression analysis showed lower odds of NTD occurrence among CC carriers, although this association was not statistically significant (OR = 0.654, 95% CI: 0.284–1.498, *p* = 0.317) as shown in Table 5.

Similarly, for rs1051266 (80A>G), the frequency of the homozygous variant genotype (GG) was also lower among cases (11.1%) compared to controls (18.2%). The recessive model analysis (GG versus AA + AG) indicated reduced odds of NTD occurrence among GG carriers, but again this finding did not reach statistical significance (OR = 0.563, 95% CI: 0.236–1.340, *p* = 0.194).

Overall, the recessive model analysis did not demonstrate a statistically significant association between maternal homozygosity for the variant alleles of either SNPs and NTD-affected births.

### 3.6. Sensitivity Analysis

Sensitivity analysis was performed using multivariable logistic regression models adjusted for maternal age and educational status (Table 6). Under the recessive genetic model, the adjusted estimates remained consistent with the unadjusted results for both *RFC1* polymorphisms.

For rs1131596, the adjusted odds ratio (AOR) for the CC genotype compared to TT + TC was 1.33 (95% CI: 0.54–3.27; *p* = 0.530), compared to an unadjusted OR of 0.65 (95% CI: 0.28–1.50; *p* = 0.317).

Similarly, for rs1051266, the adjusted odds ratio for the GG genotype compared to AG + AA was 1.26 (95% CI: 0.47–3.36; *p* = 0.649), compared to an unadjusted OR of 0.56 (95% CI: 0.24–1.34; *p* = 0.194).

Although slight changes in the magnitude and direction of the odds ratios were observed after adjustment, none of the associations reached statistical significance. These findings indicate that the results are robust and not materially influenced by the assessed confounding variables.

## 4. Discussion

In the present study, the association of *RFC1* polymorphism (rs1131596 and rs1051266) was assessed in mothers who had prior NTD births and those with no prior NTDs births. The association of these polymorphisms with socio-demographic, clinical and nutritional risk factors was assessed. We found no statistically significant association between maternal *RFC1* rs1131596 and rs1051266 polymorphisms and NTD-affected births, consistent with findings reported by some studies in the literature [21,22,23,24,25,26].

Nonetheless, this study provides important genetic data from Ethiopia, a population with one of the highest reported neural tube defect burdens globally and where genetic studies of folate metabolism pathways remain scarce.

We originally hypothesized that genetic variation in either of these two *RFC1* polymorphisms would be associated with a greater risk of offspring NTDs. However, no significant association between these two polymorphisms in the mother and NTDs in the infant were found. The rs1051266 variant is the most studied *RFC1* polymorphism. It has been associated with an increased risk of NTDs in some populations, although results vary across populations and studies [21,23,24].

A comprehensive meta-analysis by Zhang and colleagues [24], which evaluated 85 case-control comparisons across five common genetic variants, reported a suggestive but not definitively confirmed association between *RFC1* polymorphisms and an increased risk of NTDs. Specifically, in their sub-analysis of 42 studies regarding the *RFC1* 80A>G (rs1051266) variant, a potential link to NTD susceptibility was identified; however, this association was confirmed only within Asian populations. The lack of significance in other groups was attributed to the contradictory results frequently observed in studies involving Caucasian populations. These findings align with our current results, which also revealed no significant association between the *RFC1* 80A>G genotypes and NTD births (*p* = 0.605), further underscoring the population-specific nature of this genetic risk factor.

However, in contrast, a study by Cao and colleagues [27] reported a statistical difference in the allele and genotype frequencies of rs1051266 in *RFC1* gene between cases and controls and the risk for NTDs was also higher in children with G allele and GG genotype, compared with A allele and AA genotype, respectively [27]. Some researchers have suggested that the G allele is associated with reduced folate transport efficiency, leading to lower intracellular folate levels [21,26]. This folate deficiency can impair nucleotide biosynthesis and methylation reactions, which are critical for neural tube closure during embryogenesis. Similarly, a study conducted on the Han population in Northern China reported a significant correlation between a *RFC1* polymorphism (rs1051266) and increased NTD risk, particularly implicating the G allele [27].

Similarly, the rs1051266 polymorphism was associated with NTD susceptibility in both Italian [21] and Polish populations but not in Irish populations or in at least some Ethiopian population groups [25], suggesting that the genetic influence of *RFC1* polymorphisms or additional factors may vary across different populations. This health impact associated with rs1051266 is reportedly due to Arg27His amino acid substitution that occurs when the G allele is substituted by A in an *RFC1* polymorphism and may impair folate transport efficiency. The A allele (His27) is associated with reduced folate transport activity, potentially leading to lower intracellular folate levels and elevated homocysteine, a known teratogen that disrupts endothelial function and contributes to neural tube defects. Additionally, the Arg-to-His substitution may affect *RFC1* protein stability and membrane localization, further reducing folate uptake and increasing NTD risk [21].

There are also a few studies indicating increased risk of congenital anomalies like NTD births [27] as well as orofacial and conotruncal heart defects in the presence of *RFC1* A80G polymorphism [14]. These divergent results may indicate gene–nutrient interactions, variation in ethnicity, sample size limitations among cases and controls, or methodological difference across studies.

Unlike the rs1051266 SNP in *RFC1*, the effect of rs1131596 polymorphism has not to our knowledge, been studied in relation to NTDs. However, this SNP located in the SLC19A1 gene and encoding the *RFC1* protein has shown variable associations with several other disease conditions like recurrent pregnancy loss [28], leukemia [24], ischemic stroke, silent brain infarction [29] and oral cavity cancers. Notably, rs1051266 (A80G), a missense variant altering the protein sequence (His > Arg), is in strong linkage disequilibrium (LD) with rs1131596 (r^2^ = 0.98) [29]. Thus, any observed associations in other studies on rs1131596 may stem from its LD with rs1051266. The indirect implication of rs1131596 in folate metabolism, through its linkage to rs1051266, suggests a potential association with NTDs. While direct evidence linking rs1131596 to NTDs is lacking, its role in altering folate metabolism provides a plausible basis for its potential contribution to NTD births.

In our study, maternal age, education, and household economic status had no significant association with neural tube defects (NTDs), aligning with findings from local case–control studies in Eastern Ethiopia [30] and at Debre Berhan Specialized Hospital [31], which reported no significant relationships after adjusting for confounders. These results are also consistent with evidence from a large multi-centered U.S. case–control study [32], which found no association between socioeconomic factors, including maternal age, education, and income, with the occurrence of NTDs. However, some of the studies in a meta-analysis showed contradictory results, i.e an increased risk of congenital anomalies like NTD to women living in deprived neighborhoods measured according to income, education and occupation [33]. These findings underscore the multifactorial nature of NTD births with genetic predispositions, folate metabolism disruptions, and environmental factors playing more decisive roles than demographic variables.

Although the primary analyses were conducted using unadjusted logistic regression models, based on the biological premise that genotypes are fixed at conception and are generally independent of most environmental and demographic factors, we further performed a sensitivity analysis to evaluate the potential influence of key baseline characteristics. Multivariable logistic regression models adjusted for maternal age and educational status yielded results consistent with the unadjusted analyses under the recessive genetic model for both *RFC1* polymorphisms. The findings from our sensitivity analysis were consistent with the unadjusted results, further supporting the multifactorial etiology of NTDs in the Ethiopian context. This observation aligns with recent regional meta-analyses which emphasize that while genetic predispositions are critical, they operate within a complex framework of nutritional and environmental factors [33].

While minor variations in the magnitude and direction of the effect estimates were observed, none reached statistical significance, and the overall interpretation remained unchanged. These findings support the strength of the unadjusted models and suggest that the assessed baseline characteristics did not materially confound the observed associations. Nonetheless, this should be considered when interpreting the results, and future studies with larger sample sizes may enable more comprehensive adjusted analyses incorporating additional relevant factors.

The expected HWE was not observed in the control group for either *RFC1* polymorphisms. Thus, these results should be interpreted with caution, because case–control studies assume the existence of HWE at least in controls. Several factors may contribute to such deviations, including population stratification, selection bias, small sample size, and potential genotyping error [34]. In the present study, the Ethiopian population is characterized by substantial genetic diversity and possible substructure, which may lead to non-random genotype distributions. In addition, the hospital-based recruitment of controls may have introduced selection bias.

To minimize the likelihood of technical artifacts, rigorous genotyping quality-control measures were implemented, including adherence to standardized laboratory protocols, use of appropriate controls, and repeat genotyping of a subset of samples to ensure reproducibility. These steps reduce the likelihood that the observed deviation is due to genotyping error.

Although no significant association was observed, this study provides important genetic epidemiological data from Ethiopia, a population with one of the highest reported neural tube defect burdens globally [7] and where genetic studies of folate metabolism pathways remain scarce. The relatively small sample size, particularly the limited number of cases, may have reduced the statistical power of the study to detect modest genetic effects. As a result, the absence of statistically significant associations should be interpreted with caution and not necessarily as evidence of no effect. Larger studies are needed to validate these findings and provide more precise estimates of the association between *RFC1* polymorphisms and NTD births.

This study provides important insights into the genetic epidemiology of neural tube defects in an underrepresented population. To our knowledge, this is among the first to investigate *RFC1* polymorphisms rs1131596 and rs1051266 in an Ethiopian population. The findings contribute novel data on allele frequencies and genotype distributions in this setting and highlight the potential variability of genetic associations across different populations. Given the role of *RFC1* in folate transport and metabolism, these results add to the growing body of evidence exploring genetic susceptibility to NTDs and underscore the need for population-specific investigations.

### 4.1. Strengths of the Study

This study has several notable strengths. The case–control design is appropriate for investigating NTDs, which are relatively rare but carry substantial morbidity and mortality, allowing efficient comparison between affected and unaffected births. The use of robust molecular techniques, including PCR and genomic sequencing, enhanced the accuracy of polymorphism detection and minimized genotyping error.

Although no significant association was observed between NTDs and the selected *RFC1* polymorphism, the study identified a high frequency and diversity of genetic variants, providing important baseline evidence of genetic heterogeneity that may contribute to NTD births. Notably, this is the first study in Ethiopia to examine these polymorphisms using maternal whole-blood DNA, adding novel and valuable data to the local genetic research landscape.

Conducting the study in Ethiopia, a country with one of the highest (104.0 per 10,000 births) [7] reported burdens of NTD-related mortality globally, further strengthens its public health relevance. Studying genetic polymorphisms in such a high-burden context enhances the relevance of the findings and supports the need for continued genetic and nutritional research to inform prevention strategies.

### 4.2. Limitations of the Study

This study has some limitations that should be considered when interpreting the findings. First, the relatively small sample size, particularly the limited number of cases compared with controls, may have reduced the statistical power to detect modest genetic associations. In addition, while the present study focused on selected polymorphisms using targeted molecular approaches, future studies incorporating larger sample sizes and whole-genome or exome sequencing would allow a more comprehensive assessment of genetic variation and may better capture the complex genetic architecture underlying NTDs.

## 5. Conclusions

Although this study did not identify a statistically significant association between maternal *RFC1* gene polymorphisms and the occurrence of NTD-affected births, the findings do not eliminate the possibility of a genetic contribution. Given the complex and multifactorial nature of NTDs, the role of *RFC1* variants may be subtle or context-dependent, requiring more sensitive or comprehensive approaches to detect.

To gain a more complete understanding of the genetic architecture underlying NTDs in the Ethiopian population, further investigations are necessary. Advanced genomic techniques such as whole-genome sequencing of *RFC1* and other genes involved in folate metabolism could help identify rare or interacting variants that contribute to NTD susceptibility. Such efforts are crucial for informing future prevention strategies and tailoring interventions to high-risk groups.

## Figures and Tables

**Table 1:** Socio-demographic characteristics of mothers with (cases) and without (controls) a prior NTD birth.

Characteristics	Cases (*n* = 63)	Controls (*n* = 187)	Total (*n* = 250)	χ^2^	*p*-Value
Maternal age (years)				1.34	0.935
16–20	1 (1.6%)	3 (1.6%)	4 (1.6%)		
20–34	51 (81.0%)	155 (82.9%)	206 (82.4%)		
35–45	11 (17.5%)	29 (15.5%)	40 (16.0%)		
Residence				12.40	<0.001
Urban	51 (81.0%)	178 (95.2%)	229 (91.6%)		
Rural	12 (19.0%)	9 (4.8%)	21 (8.4%)		
Marital status				1.33	0.249
Unmarried	2 (3.2%)	2 (1.1%)	4 (1.6%)		
Married	61 (96.8%)	185 (98.9%)	246 (98.4%)		
Educational status				5.53	0.137
No formal education	7 (11.1%)	13 (7.0%)	20 (8.0%)		
Primary school	23 (36.5%)	70 (37.4%)	93 (37.2%)		
Secondary school	30 (47.6%)	76 (40.6%)	106 (42.4%)		
Higher education and above	3 (4.8%)	28 (15.0%)	31 (12.4%)		
Occupation				47.41	<0.001
Housewife	62 (98.4%)	93 (49.7%)	155 (62.0%)		
Employed/business	1 (1.6%)	94 (50.3%)	95 (38.0%)		
Monthly income				26.70	<0.001
Low	40 (63.5%)	51 (27.3%)	91 (36.4%)		
Lower-middle	23 (36.5%)	136 (72.7%)	159 (63.6%)		
Religion				4.52	0.341
Orthodox	48 (76.2%)	136 (72.7%)	184 (73.6%)		
Muslim	13 (20.6%)	29 (15.5%)	42 (16.8%)		
Protestant	2 (3.2%)	20 (10.7%)	22 (8.8%)		
Number of children				1.21	0.750
One	35 (55.6%)	114 (61.0%)	149 (59.6%)		
Two	24 (38.1%)	58 (31.0%)	82 (32.8%)		
≥Three	4 (6.4%)	15 (8.0%)	19 (7.6%)		

**Table 2:** Hardy–Weinberg equilibrium analysis of *RFC1* SNPs among case and control mothers.

SNP	Genotype	Cases (*n* = 63) Observed	Expected	Controls (*n* = 187) Observed	Expected	χ^2^ (Case)	χ^2^ (Control)	*p*-Value (Case)	*p*-Value (Control)
rs1131596 (−43T>C)	TT	48 (76.2%)	42.36	134 (71.6%)	108.01	0.75	6.25	<0.001	<0.001
	TC	7 (11.1%)	18.59	19 (10.2%)	68.21	7.23	35.50		
	CC	8 (12.7%)	2.04	34 (18.2%)	10.77	17.41	20.07		
	**Total χ^2^**					25.39	61.82		
rs1051266 (80A>G)	AA	36 (57.1%)	33.58	103 (55.1%)	87.62	0.18	2.55	0.127	<0.001
	AG	20 (31.7%)	24.82	50 (26.7%)	80.76	0.94	11.06		
	GG	7 (11.1%)	4.60	34 (18.2%)	18.62	0.52	3.96		
	**Total χ^2^**					1.64	18.74		

**Table 3:** Allele frequency of selected *RFC1* SNPs of the Ethiopian mothers compared to the average for the African population according to Allele Frequency Aggregator (ALFA).

SNP	Population	Group	Reference Allele	Alternate Allele
rs1131596 (−43T>C)	Ethiopia (*n* = 250)	Cases	G = 0.818	A = 0.182
		Controls	G = 0.767	A = 0.232
	Africa (*n* = 1322)	—	G = 0.719	A = 0.280
rs1051266 (80A>G)	Ethiopia (*n* = 250)	Cases	T = 0.762	C = 0.238
		Controls	T = 0.685	C = 0.315
	Africa (*n* = 1322)	—	T = 0.673	C = 0.327

**Table 4:** Distribution of rs1131596 & rs1051266 genotypes and alleles among women with and without a prior NTD birth using additive model.

SNP/Genotype	Total (*n* = 250)	Cases (*n* = 63)	Controls (*n* = 187)	*p*-Value	OR	95% CI
rs1131596 (−43T>C)						
TT (Reference)	182 (72.8%)	48 (76.2%)	134 (71.7%)	0.605	—	—
TC	26 (10.4%)	7 (11.1%)	19 (10.2%)	0.953	1.029	0.407–2.599
CC (Alternate)	42 (16.8%)	8 (12.7%)	34 (18.2%)	0.325	0.657	0.284–1.518
C allele	110 (22.0%)	23 (18.2%)	87 (23.2%)	—	—	—
T allele	390 (78.0%)	103 (81.8%)	287 (76.7%)	—	—	—
rs1051266 (80A>G)						
AA (Reference)	139 (55.6%)	36 (57.1%)	103 (55.1%)	0.425	—	—
AG	70 (28.0%)	20 (31.7%)	50 (26.7%)	0.681	1.144	0.602–2.176
GG (Alternate)	41 (16.4%)	7 (11.1%)	34 (18.2%)	0.248	0.589	0.240–1.445
A allele	313 (72.8%)	94 (76.2%)	256 (68.5%)	—	—	—
G allele	117 (27.2%)	34 (23.8%)	118 (31.5%)	—	—	—

Odds ratios (ORs) and 95% confidence intervals (CIs) represent crude associations between genotype categories and NTD outcome.

**Table 5:** Genotypes and Alleles for selected SNPs of *RFC1* variant (rs1131596 & rs1051266) among women with and without a prior NTD birth using recessive model.

Genotype (Recessive Model)	Total (*n* = 250)	Cases (*n* = 63)	Controls (*n* = 187)	*p*-Value	OR	95% CI
rs1131596 (−43T>C)						
CC (Alternate)	42 (16.8%)	8 (12.7%)	34 (18.2%)	0.317	0.654	0.284–1.498
TT + TC	208 (83.2%)	55 (87.3%)	153 (81.9%)	—	—	—
rs1051266 (80A>G)						
GG (Alternate)	41 (16.4%)	7 (11.1%)	34 (18.2%)	0.194	0.563	0.236–1.340
AG + AA	209 (83.6%)	56 (88.8%)	153 (81.8%)	—	—	

Odds ratios (ORs) and 95% confidence intervals (CIs) represent crude associations between genotype categories and NTD outcome.

**Table 6:** Sensitivity Analysis of *RFC1* Polymorphisms (Recessive Model).

SNP	Model	OR	95% CI	*p*-Value
rs1131596 (−43T>C)				
CC vs. (TT + TC)	Unadjusted	0.654	0.284–1.498	0.317
	Adjusted *	1.333	0.543–3.267	0.530
rs1051266 (80A>G)				
GG vs. (AG + AA)	Unadjusted	0.563	0.236–1.340	0.194
	Adjusted *	1.256	0.470–3.358	0.649

* Adjusted for maternal age and educational status.

## Data Availability

Ethical and legal restrictions specified in the approved protocol preclude unrestricted public deposition of the individual-level data. De-identified data, including chromatograms, and summary statistics not presented in the manuscript may be made available upon reasonable request to the first author, contingent on approval by the relevant ethics committee and the execution of a data-transfer agreement. Requests should be directed to the first author and will be evaluated on a case-by-case basis.

## Abbreviations

The following abbreviations are used in this manuscript:

AAU	Addis Ababa University
ALFA	Allele Frequency Aggregator
EPHI	Ethiopian Public Health Institute
HWE	Hardy–Weinberg equilibrium
ICD	International Classification of Diseases
MAF	Mean Allele Frequency
MTHFR	Methylenetetrahydrofolate Reductase
NCBI	National Center for Biotechnology Information
NTD	Neural Tube Defect
ODK	Open Data Kit
OSU	Oklahoma State University
PCR	Polymerase Chain Reaction
RFC	Reduced Folate Carrier
SNP	Single Nucleotide Polymorphism
